# Evaluating the Diagnostic Accuracy of a Novel Bayesian Decision-Making Algorithm for Vision Loss

**DOI:** 10.3390/vision6020021

**Published:** 2022-04-04

**Authors:** Amy Basilious, Chris N. Govas, Alexander M. Deans, Pradeepa Yoganathan, Robin M. Deans

**Affiliations:** 1 Schulich School of Medicine and Dentistry, Western University, 1151 Richmond St., London, ON N6A 5C1, Canada; amybasilious@gmail.com (A.B.); adeans2@uwo.ca (A.M.D.); 2 School of Medicine, Ross University, Two Mile Hill, St. Michael, Bridgetown BB11093, Barbados; chrisgovas@mail.rossmed.edu; 3 Department of Ophthalmology, Kresge Eye Institute, Wayne State University School of Medicine, Wayne State University, 540 E. Canfield Ave., Detroit, MI 48201, USA; deepayoganathan@gmail.com; 4 Windsor Eye Associates, Department of Ophthalmology and Vision Sciences, University of Toronto, 2224 Walker Rd #198, Windsor, ON N8W 3P6, Canada; 5 Department of Ophthalmology, Schulich School of Medicine and Dentistry, Western University, 1151 Richmond St., London, ON N6A 5C1, Canada

**Keywords:** algorithm, diagnosis, differential, vision disorder, vision loss

## Abstract

The current diagnostic aids for acute vision loss are static flowcharts that do not provide dynamic, stepwise workups. We tested the diagnostic accuracy of a novel dynamic Bayesian algorithm for acute vision loss. Seventy-nine “participants” with acute vision loss in Windsor, Canada were assessed by an emergency medicine or primary care provider who completed a questionnaire about ocular symptoms/findings (without requiring fundoscopy). An ophthalmologist then attributed an independent “gold-standard diagnosis”. The algorithm employed questionnaire data to produce a differential diagnosis. The referrer diagnostic accuracy was 30.4%, while the algorithm’s accuracy was 70.9%, increasing to 86.1% with the algorithm’s top two diagnoses included and 88.6% with the top three included. In urgent cases of vision loss (*n* = 54), the referrer diagnostic accuracy was 38.9%, while the algorithm’s top diagnosis was correct in 72.2% of cases, increasing to 85.2% (top two included) and 87.0% (top three included). The algorithm’s sensitivity for urgent cases using the top diagnosis was 94.4% (95% CI: 85–99%), with a specificity of 76.0% (95% CI: 55–91%). This novel algorithm adjusts its workup at each step using clinical symptoms. In doing so, it successfully improves diagnostic accuracy for vision loss using clinical data collected by non-ophthalmologists.

## 1. Introduction

Diagnoses of acute vision loss often pose significant challenges for healthcare providers [1]. Diagnostic uncertainty causes the suboptimal triaging of cases, leading to unnecessary tests and referrals and delayed patient care [2,3].

Clinicians are natural Bayesian decision makers. They use known history and physical exam results (pre-test odds) to adjust the probability of a given disease and infer the next appropriate workup step [4]. Current resources available to the non-ophthalmologist, such as UpToDate and clinical practice guidelines, have limitations. While UpToDate provides encyclopedic and comprehensive documentation on many clinical disorders, it fails to provide easy, stepwise workups [5]. Clinical practice guidelines use static algorithms (traditional flowcharts) to provide general approaches [6]. However, static algorithms do not account for pre-test probability when suggesting the next steps. They require users to ask the same series and number of questions to obtain a diagnosis, rendering them inflexible and inefficient [7,8].

The field of medical diagnostics is being transformed by electronic aids that utilize machine learning and artificial intelligence. Within the field of ophthalmology, these tools have made it possible to automate the detection of diabetic retinopathy and glaucoma from retinal fundus imaging and the detection of choroidal neovascularization from Ocular Coherence Tomography (OCT) images [9,10,11]. These valuable electronic aids have the potential to improve triaging for patients who have already been referred to specialists [12]. These tools largely focus on diagnostics based on imaging and do not take symptoms and signs into account. There is a paucity of clinical decision support resources to aid general practitioners and emergency department staff at point of care.

A robust, dynamic algorithm that simplifies and communicates a clear medical workup has the potential to fill this gap in the available diagnostic aids. The authors (AD and RD) developed Pickle, a novel app that uses Bayesian algorithms to provide primary care practitioners with appropriate workups for common ophthalmic presentations. The algorithms employ a dynamic Bayesian feedback process to recreate themselves continuously at each step of decision making.

This study tested the acute vision loss algorithm of the Pickle app by comparing its diagnostic accuracy to that of referring and specialist physicians. Similar early intervention diagnostic decision-support systems for primary care practitioners have been shown to improve diagnostic accuracy, eliminate unnecessary tests and referrals, and decrease wait times [13,14,15,16].

## 2. Materials and Methods

### 2.1. Pickle App Design and Development

**Figure 1:**
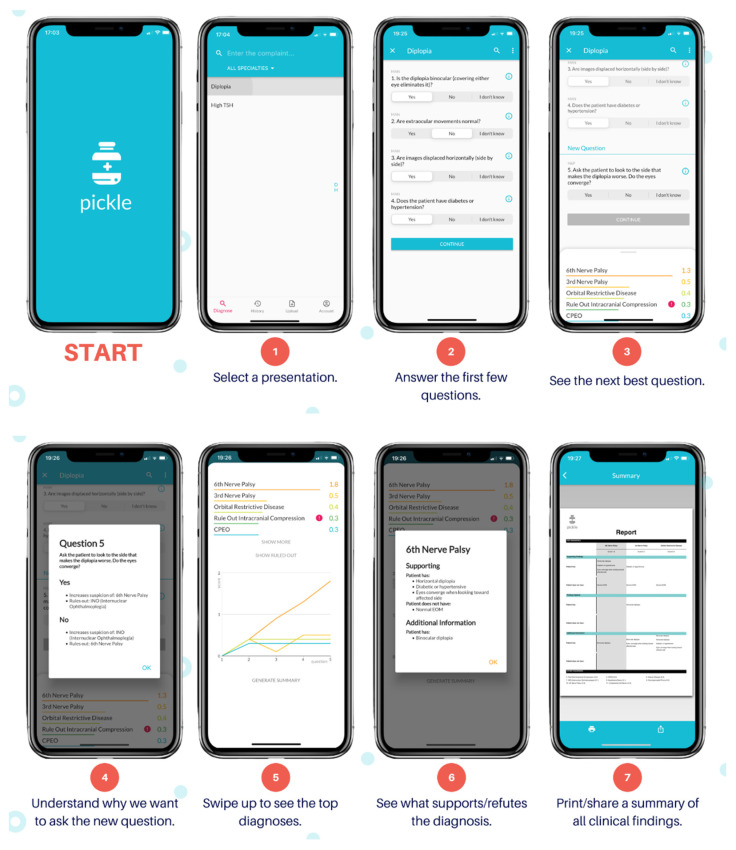
Pickle app user interface.

The authors (AD and RD) developed Pickle, a novel app that employs a dynamic Bayesian feedback process. Figure 1 shows the Pickle app user interface. Pickle prompts the user with 3–4 initial “Yes/No/I don’t know” questions to begin narrowing the differential diagnosis. It then provides a differential diagnosis ranked by likelihood according to the user’s answers. Subsequently, Pickle employs a proprietary Bayesian-grounded artificial intelligence program to provide the most appropriate next step for the workup. Specifically, the most recent differential diagnosis is analyzed to determine which remaining questions can rule out diagnoses, markedly reduce their likelihood, or markedly increase their likelihood. Questions are stored against a table of values that represent their ability to stratify diseases by raising or decreasing the level of suspicion for certain diseases depending on the answer. Generally, using a Bayesian approach, the question with the highest potential difference between pre-test probability and post-test probability is asked of the user to expedite the algorithm’s decision-making. The dynamic part of the program is repeated until the user terminates the program or the level of suspicion for the top diagnosis heavily outweighs that of the others. Additionally, users can select a diagnosis within the differential to see which symptoms or history support or refute it.

The authors have currently made Pickle available for beta testing in three Ontario emergency room settings. Access will be expanded free-of-charge to all English-speaking clinicians in future months when the technological infrastructure can support them. Future algorithms may be translated and updated for use in specific geographical areas. The app will operate as a third-party diagnostic aid. If in the future it is integrated as a widget into current electronic medical records (EMRs), regulatory approval may be required, but it has not been pursued at this time. Pickle’s algorithms have the potential to improve diagnostic decisions at point of care in primary care settings, thus impacting immediate management and referral decisions.

### 2.2. Study Design and Sample

The study design tests the hypothesis that a dynamic Bayesian algorithm can improve the diagnostic accuracy of vision loss complaints when compared to that of primary care physicians, using an ophthalmologist’s diagnostic impression as a gold standard. This study was conducted in accordance with the tenets of the Declaration of Helsinki.

**Figure 2:**
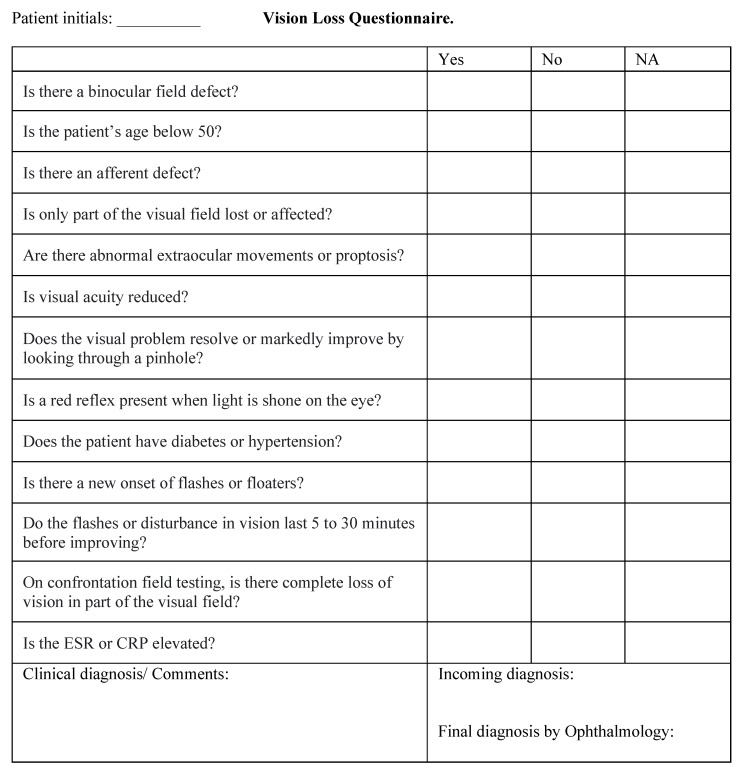
Questionnaire including all possible questions of the Pickle vision loss algorithm.

This was a prospective study. A questionnaire was developed with a list of all possible algorithm questions (Figure 2). Questions focused on a patient’s medical history and physical examination and did not necessitate fundoscopy.

The paper questionnaire was distributed to all healthcare providers (emergency departments and primary care physicians) who referred adult patients with acute vision loss to an ophthalmologist in Windsor, Ontario, Canada. Questionnaires were completed by hand and did not employ checkboxes or multiple-choice formats when prompting for the referrer’s diagnosis. Inclusion criteria comprised (i) adults above the age of 18 who (ii) presented with acute vision loss, as (iii) determined by the referring physician with (iv) no prior diagnosis for the presenting complaint. Those who presented with concurrent red eye or diplopia were not excluded. The time interval between the referrer assessment and the ophthalmologist assessment was less than one week for all patients, with no clinical interventions during this interval. A consecutive series sampling method was used, with a sample size of 79 patients. This sample size allowed for all causes of acute vision loss to be represented. Data were collected between October 2020 and March 2021.

Referrers completed the questionnaire based on the patient’s medical history and physical assessment and documented their suspected diagnosis (termed the “referrer diagnosis”). The questionnaire with the referrer diagnosis was faxed alongside a referral to the staff ophthalmologist. The ophthalmologist then assessed the patient and attributed a gold-standard diagnosis. Since the ophthalmologist received the referral, he was not blinded to the referrer diagnosis. Post-visit, questionnaire data were entered into Pickle’s vision loss algorithm to produce an algorithm differential. Questions for which no responses were documented by referrers were entered as “don’t know” on the algorithm. The ophthalmologist was blinded to the algorithm differential.

### 2.3. Algorithm Diagnoses

The algorithm may make 13 possible diagnoses for acute vision loss based on conventional ophthalmological diagnostic grouping:

1. Optic neuritis
2. Optic nerve compression
3. Non-arteritic anterior ischemic optic neuropathy (NAION)/branch retinal artery occlusion (BRAO)/branch vein occlusion (BVO)
4. Central retinal artery occlusion (CRAO)
5. Central vein occlusion (CVO)
6. Temporal arteritis
7. Other macular disease
8. Peripheral retinal issue (retinal tear or detachment)
9. Vitreous floaters/posterior vitreous detachment (PVD)
10. Vitreous hemorrhage
11. Lens/cornea issue (including acute angle glaucoma)
12. Migraine
13. Post-chiasmal disease

This list of possible diagnoses was available to the staff ophthalmologist as well as to the investigators who entered the clinical data from referrers into the algorithm.

NAION, BRAO, and BVO were assimilated into a single diagnostic group (#3) since they may present with symptoms of peripheral vision loss. This is contrasted with other macular causes of vision loss that comprise diagnostic group #7, including central serous chorioretinopathy, wet macular degeneration, macular hole, epiretinal membrane, and cystoid macular edema. This classification allows for differentiation between the two groups using only symptoms and is therefore of greater use to the non-ophthalmologist. Further differentiation between individual diagnoses within groups #3 and #7 would require ophthalmic investigation/equipment.

A list of abbreviations and their meanings are shown in Table 1.

### 2.4. Outcome Measures and Data Analysis

The referrer diagnostic accuracy was defined as the concordance between the referrer diagnosis and the gold-standard diagnosis. If no diagnosis was made by the referrer, this was considered an incorrect diagnosis. Algorithm diagnostic accuracy was defined as the concordance between the algorithm differential and the gold-standard diagnosis. Since the algorithm ranks the differential diagnoses, accuracy was assessed based on three divisions: the accuracy of the top-scoring diagnosis (Top 1), the top two scoring diagnoses (Top 2), and the top three scoring diagnoses (Top 3). For further analysis, diagnoses were grouped into 6 different clusters based on anatomical demarcations in the visual axis:

- Peripheral Retinopathy/Vitreous
- Optic Nerve/Circulation
- Other Macular Disease
- Media
- Migraine
- Post-Chiasmal Disease

The referrer and algorithm diagnostic accuracies were determined for each cluster. These measures were also determined for a subset of urgent cases. The sensitivity and specificity of the algorithm’s ability to identify cases as urgent or non-urgent were computed.

## 3. Results

Questionnaires were completed for 79 patients referred with acute vision loss between October 2020 and March 2021. All questionnaires were included in the analysis.

Based on the gold-standard diagnosis, the causes of vision loss were: vitreous hemorrhage (*n* = 13), peripheral retinal issue (*n* = 12), NAION/BRAO/BVO (*n* = 11), other macular disease (*n* = 8), lens/cornea issue (*n* = 6), vitreous floaters/PVD (*n* = 5), migraine (*n* = 6), CVO (*n* = 3), optic neuritis (*n* = 4), CRAO (*n* = 2), temporal arteritis (*n* = 3), optic nerve compression (*n* = 5), post-chiasmal disease (*n* = 2), and endophthalmitis (*n* = 1). Two cases had both a peripheral retinal issue and a vitreous hemorrhage. When clustered, there were 28 cases of Peripheral Retinopathy/Vitreous, 28 of Optic Nerve/Circulation, 8 of Other Macular Disease, 6 of Media, 6 of Migraine, 2 of Post-Chiasmal Disease, and 1 Other (endophthalmitis) (Table 2).

### 3.1. Referrer Diagnostic Accuracy

**Figure 3:**
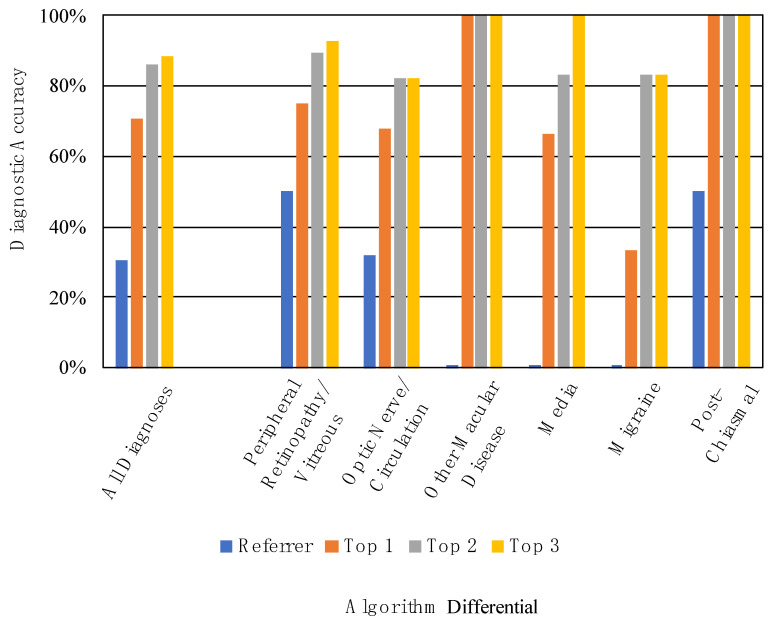
Referrer and algorithm diagnostic accuracies for all diagnoses and by diagnostic cluster.

The referrer diagnosis was correct in 30.4% (24/79) of cases (Figure 3). Thirty-seven referrals either had no attempted diagnosis or only described a sign or symptom, for example, “floaters and flashes” or “vision loss NYD” (not yet diagnosed). These were marked as incorrect. The most common referrer diagnoses were retinal detachment (*n* = 18) and stroke (*n* = 8). The referrer diagnostic accuracy was 50.0% for Peripheral Retinopathy/Vitreous (14/28), 32.1% for Optic Nerve/Circulation (9/28), 0% for Other Macular Disease (0/8), 0% for Media (0/6), 0% for Migraine (0/6), and 50.0% for Post-Chiasmal Disease (1/2) (Figure 3, Table 3).

### 3.2. Algorithm Diagnostic Accuracy

When considering only the top-scoring diagnosis of the differential, the algorithm’s diagnostic accuracy was 70.9% (56/79). When considering the top two diagnoses, accuracy was 86.1% (68/79). When considering the top three diagnoses, accuracy was 88.6% (70/79) (Figure 3). Table 3 displays the referrer and algorithm diagnostic accuracies for each of the diagnostic clusters.

### 3.3. Referrer and Algorithm Diagnostic Accuracy in Urgent Conditions

**Figure 4:**
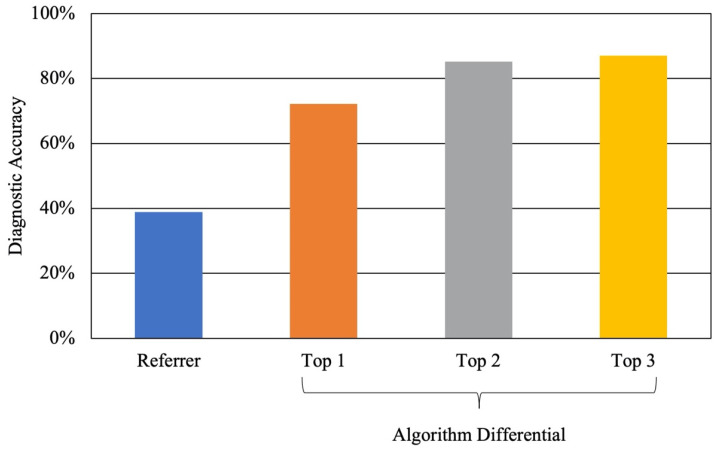
Referrer and algorithm diagnostic accuracy for urgent conditions.

Fifty-four cases were deemed “urgent conditions” needing rapid referral to consider serious pathology. These were in the following clusters: peripheral retina, vitreous hemorrhage, optic nerve, and post-chiasmal disease. The referrer diagnostic accuracy for urgent conditions was 38.9% (21/54), with 22 of these cases lacking a referrer diagnosis (Figure 4). The algorithm diagnostic accuracy for these urgent cases was 72.2% (39/54) when considering the top diagnosis. This increased to 85.2% (46/54) with the top two diagnoses and 87.0% (47/54) with the top three diagnoses. Additionally, for these urgent cases, the algorithm’s top diagnosis was correct in 22 cases that were not accurately diagnosed by referrers.

For non-urgent cases, the algorithm correctly diagnosed 68.0% of cases (17/25). The referrer sensitivity for determining the urgency of cases, regardless of the diagnosis, was 57.4% (95% CI: 43–71%). The referrer specificity for the same was 84.0% (95% CI: 64–95%). However, the algorithm sensitivity for the urgency of a case using the top diagnosis was 94.4% (95% CI: 85–99%). The algorithm specificity was 76.0% (95% CI: 55–91%).

## 4. Discussion

The baseline accuracy of referring healthcare providers was 30.4% for all cases, rising to 38.9% for urgent cases. No referring diagnosis was made in nearly half of all referrals, which may reflect a lack of confidence to attempt a diagnosis or consider a differential. Given that the questionnaires were hand-written, non-prompted (no checkboxes for diagnoses), and anonymous, it was thought that they would encourage referrers to provide their own diagnoses. Referrals were more likely to have a diagnosis when patients had flashes and floaters with normal visual acuity. A recent study of referrals to an emergency eye clinic found referral diagnostic accuracy to be 39% for emergency physicians and 33% for primary care practitioners [17]. These findings demonstrate that diagnostic decision aids would benefit non-ophthalmologists who assess patients with vision loss.

Given equivalent clinical data, the algorithm improved on the referrer diagnostic accuracy (30.4%) to a range of 70.9–88.6%. The difficulty of performing accurate direct ophthalmoscopy is clearly known [18]. Importantly, the algorithm’s questions did not require the user to conduct a fundus exam, instead querying only the presence of a red reflex.

Technological advancements are increasingly improving medical diagnostics. Available electronic aids have focused on improving diagnostics by automating image analysis [9,10,11,12]. However, most of these AI-assisted technologies are designed to identify a single disease, in contrast to our algorithm that differentiates between diagnoses [19]. Furthermore, the difficulty of detecting rare diseases using images and deep learning methods is known [20]. While these tools make a valuable contribution to care, they are not yet targeted for use by general practitioners and are not designed for acute or uncommon presentations. The Pickle algorithm simply uses a series of questions that can be answered by general practitioners in any setting to achieve the same objectives that complex machine learning methods seek to accomplish using fundus photographs, anterior segment photographs, and OCTs [21,22]. The presented algorithm and Pickle app serve to fill the gap in available diagnostic aids, providing a practical approach to acute vision loss. A previously published study in the UK was the first to test the diagnostic accuracy of a static algorithm for vision loss [8]. The algorithm improved diagnostic accuracy from 51% to 84%. The novelty of the presented Pickle algorithm lies in its dynamic nature: it adjusts the sequence of each workup as a patient’s clinical findings are entered. Additionally, the algorithm produces a differential, whereas static algorithms produce a single diagnosis. The differential better reflects clinical practice. Importantly, it ensures that the end user continues to consider critical diagnoses, even if they are not the most likely diagnosis.

An ideal algorithm-based tool would be highly sensitive to the most urgent conditions (peripheral retina, optic nerve, and post-chiasmal pathology) while maintaining a high specificity for less urgent conditions (macula, media, and migraine). This would allow the physician to diagnose or refer more confidently. For urgent conditions, the algorithm’s top diagnosis had a sensitivity of 94.4%. This was expected, as the algorithm was designed to consider the most urgent conditions first. If clinical findings are unable to rule out urgent conditions, the algorithm retains them in the differential to alert the clinician. Nearly half of patients with urgent conditions had no referring diagnosis attempted (22/54). Of these patients, 19 of 22 were correctly diagnosed by the Pickle algorithm, thus facilitating more appropriate assessment and triaging.

In nine cases, Pickle’s top-three differential did not contain the gold-standard diagnosis. In the first case, the diagnostic error was caused by the failure of the clinician to assess for an afferent pupillary defect, despite prompting by the questionnaire. The algorithm subsequently defaulted to a worst-case scenario in an older patient with vision loss. It suggested a differential containing a stroke of the optic nerve, temporal arteritis, and macular disease. This patient had a longstanding retinal detachment with vision loss. In a second case, a younger patient under the age of 50 presented with an idiopathic branch retinal artery occlusion (stroke). Typically, in younger patients without a history of diabetes or hypertension, the differential for an optic nerve disorder should point towards optic neuritis and optic nerve compression, as a stroke is less likely. In four other cases diagnosed by ophthalmology as optic neuropathy, NAION, and BRVO, the referrer indicated ‘no red reflex’. Acute vision loss with an absent red reflex suggests a vitreous hemorrhage, which the algorithm appropriately identifies as the likely cause. It also rules out optic neuropathy and circulation problems, which should have an intact red reflex. Two other cases can be explained by errors in completing the questionnaire: (1) a patient was referred with a diagnosis of “floaters”, but the questionnaire answers indicated no flashes or floaters; and (2) a patient was ultimately diagnosed with a migraine, but the referrer indicated a binocular field defect. The algorithm appropriately identified a posterior chiasm problem as the most likely cause of a binocular field defect. In the last case, “endophthalmitis” was not diagnosed by the algorithm, as this diagnosis is not on the algorithm’s differential. However, the algorithm correctly identified the problem as arising in the vitreous. We will change the diagnosis in the algorithm from “vitreous hemorrhage” to “vitreous problem” to include other causes of acute vision loss arising from the vitreous, such as inflammation.

### Limitations

A limitation of this study is the small sample size of patients presenting with vision loss due to media, migraines, and post-chiasmal disease. Post-chiasmal disease is an uncommon presentation, therefore leading to a small sample size. For this reason, our analyses of the referrer and algorithm accuracies for these presentations were limited. Another limitation is that only patients referred for specialist care were included in the study. However, we expect these patients to act as a representative sample of the target population. This is because most patients presenting with vision loss are referred onwards, as recommended by the Canadian Ophthalmological Society and the American Academy of Ophthalmology [23,24].

Other minor limitations are user error and atypical presentations. Incorrect physical examination techniques or non-responses may lead to inaccurate diagnoses. In addition, the correct diagnosis may be ranked lower if presenting atypically. However, this mirrors clinical practice, in which atypical presentations of vision loss often require further investigation to elucidate their cause. Importantly, in cases of user non-responses or atypical presentations, the algorithm errs on the side of caution by retaining urgent conditions in the differential. Furthermore, previous research has found that diagnostic performance decreases as the number of target diagnoses increases [25]. Fewer and broader diagnostic categories may have improved the diagnostic accuracy of the algorithm. However, this would have decreased the app’s utility for advising referral decisions.

## 5. Conclusions

Healthcare professionals in primary care settings and emergency departments are often the first point of contact for patients with vision loss. This research found a referrer diagnostic accuracy of 30.4%, demonstrating that acute vision loss presents diagnostic challenges in these settings. We have shown that Pickle’s Bayesian algorithms successfully improve diagnostic accuracy in these cases to a range of 70.9–88.6%. The algorithm’s high sensitivity in urgent cases is particularly impactful, as nearly half of these cases were found to have no referrer diagnosis attempted. Furthermore, it yields this benefit using only the clinical tools available to non-ophthalmologists, without requiring fundoscopic findings. This novel diagnostic aid should be used as an adjunct to clinical judgement in primary care settings to help optimize patient outcomes. Improvements in diagnostic accuracy may allow for better patient triaging, potentially reducing unnecessary utilization of resources and expediting care in critical cases.

## Figures and Tables

**Table 1:** Abbreviations.

Abreviation	Meaning
BRAO	Branch retinal artery occlusion
BVO	Branch vein occlusion
CRAO	Central retinal artery occlusion
CVO	Central vein occlusion
NAION	Non-arteritic ischemic optic neuropathy
PVD	Posterior vitreous detachment

**Table 2:** Number of individual diagnoses per diagnostic cluster. Abbreviations: BRAO (branch retinal artery occlusion), BVO (branch vein occlusion), CRAO (central retinal artery occlusion), CVO (central vein occlusion), NAION (non-arteritic ischemic optic neuropathy), PVD (posterior vitreous detachment).

Diagnostic Cluster	Diagnoses
Peripheral Retinopathy/Vitreous (*n* = 28) ^1^	Vitreous hemorrhage (*n* = 13) Peripheral retinal issue (*n* = 12) Vitreous floaters/PVD (*n* = 5)
Optic Nerve/Circulation (*n* = 28)	NAION/BRAO/BVO (*n* = 11) CVO (*n* = 3) Optic neuritis (*n* = 4) CRAO (*n* = 2) Temporal arteritis (*n* = 3) Optic nerve compression (*n* = 5)
Other Macular Disease (*n* = 8)	Other macular disease (*n* = 8)
Media (*n* = 6)	Lens/cornea issue (*n* = 6)
Migraine (*n* = 6)	Migraine (*n* = 6)
Post-Chiasmal (*n* = 2)	Post-chiasmal disease (*n* = 2)
Other (*n* = 1)	Endophthalmitis (*n* = 1)

^1^ Accounting for two patients with both a peripheral retinal issue and a vitreous hemorrhage.

**Table 3:** Referrer and algorithm diagnostic accuracies for each diagnostic cluster.

Diagnostic Cluster	Diagnostic Accuracy			
		Algorithm Differential		
	Referrer Diagnosis	Top Diagnosis	Top 2 Diagnoses	Top 3 Diagnoses
Peripheral Retinopathy/Vitreous (*n* = 28)	14/28	21/28	25/28	26/28
Optic Nerve/Circulation (*n* = 28)	9/28	19/28	23/28	23/28
Other Macular Disease (*n* = 8)	0/8	8/8	8/8	8/8
Media (*n* = 6)	0/6	4/6	5/6	6/6
Migraine (*n* = 6)	0/6	2/6	5/6	5/6
Post-Chiasmal (*n* = 2)	1/2	2/2	2/2	2/2
Other (*n* = 1)	0/1	0/1	0/1	0/1
Total	24/79	56/79	68/79	70/79

## Data Availability

The data presented in this study are available in the article.
